# WES data from 286 diffuse gliomas under the 2021 WHO Classification of Tumors of the Central Nervous System

**DOI:** 10.1038/s41597-022-01823-3

**Published:** 2022-11-11

**Authors:** Zheng Zhao, Ke-Nan Zhang, Zhiyan Sun, Changlin Yang, Qiangwei Wang, Guanzhang Li, Zhiliang Wang, Fan Zeng, Ruichao Chai, Zenghui Qian, Zheng Wang, Yanwei Liu, Wenping Ma, Fan Wu, Tao Jiang

**Affiliations:** 1 Beijing Neurosurgical Institute, Capital Medical University, 100070 Beijing, China; 2 Department of Neurosurgery, The Second Affiliated Hospital of Zhejiang University School of Medicine, Hangzhou, 310009 China; 3 Department of Neurosurgery, Beijing Tiantan Hospital, Capital Medical University, 100070 Beijing, China; 4 Department of Radiotherapy, Beijing Tiantan Hospital, Capital Medical University, 100070 Beijing, China; 5 Department of Neurosurgery, Beijing Children’s Hospital, Capital Medical University, National Center for Children’s Health, 100045 Beijing, China; 6 Center of Brain Tumor, Beijing Institute for Brain Disorders, 100069 Beijing, China; 7 China National Clinical Research Center for Neurological Diseases, 100070 Beijing, China; 8 Research Unit of Accurate Diagnosis, Treatment, and Translational Medicine of Brain Tumors, Chinese Academy of Medical Sciences, 100070 Beijing, China

**Keywords:** CNS cancer, Genetics research, Cancer genomics, Cancer genetics

## Abstract

Diffuse gliomas (DGs) are the most common and lethal primary neoplasms in the central nervous system. The latest 2021 World Health Organization (WHO) Classification of Tumors of the Central Nervous System (CNS) was published in 2021, immensely changing the approach to diagnosis and decision making. As a part of the Chinese Glioma Genome Atlas (CGGA) project, our aim was to provide genomic profiling of gliomas in a Chinese cohort. Two hundred eighty six gliomas with different grades were collected over the last decade. Using the Illumina HiSeq platform, over 75.8 million high-quality 150 bp paired-end reads were generated per sample, yielding a total of 43.4 billion reads. We also collected each patient’s clinical and pathological information and used it to annotate their genetic data. All patients were diagnosed and classified by neuro-pathologist under the 2021 WHO classification. This dataset provides an important reference for researchers and will significantly advance our understanding of gliomas.

## Abstract (summary)

**Table:** 

Measurement(s)	glioma
Technology Type(s)	Whole Exome Sequencing
Sample Characteristic - Organism	Homo sapiens

## Background & Summary

Diffuse gliomas (DGs) are the most common and lethal type of primary neoplasm in the central nervous system and are the leading cause of cancer death in adolescents and young adults (AYAs)^1,2^. In part due to the disadvantages in historical classification, standard treatment provided limited benefit^3^. Patients survival ranged from 6 months to decades^4^. Neuropathologists have been devoted to classifying diffuse gliomas more precisely to predict survival and guide treatment strategies for decades and have gradually confirmed the significance and rationality of incorporating molecular characteristics into the classification strategy^5,6^.

The cancer genome atlas (TCGA) and the Rembrandt Project (REpository for Molecular BRAin Neoplasia DaTa) contain thousands of glioma specimens and genomic data^7–9^, but the included patients had incomplete molecular characterization. Asian patients were also severely underrepresented. In 2005, the Chinese Glioma Genome Atlas (CGGA) project began to enroll patients, collect tissue specimens, conduct multi-omics sequencing, and finally developed an online portal in 2020^10^.

Here, as a part of the CGGA project, we provide a whole-exome sequencing (WES) dataset with molecular biomarker information classified under the 2021 WHO Classification of Tumors of the Central Nervous System^11^, which depicts the genomic landscape of DGs under the new classification. Encouragingly, this dataset is the largest public WES dataset from the Chinese DG cohort.

In this work, the WES libraries were sequenced using the Illumina HiSeq platform. Approximately 43.4 billion 150-bp paired-end reads were generated, with an average of over 75.8 million sequence reads per sample. For each sample, we first aligned the raw reads to the reference human genome. Then, we developed a computational pipeline to identify glioma-associated somatic mutations to catalogue the genomic mutational profiles of the cohort of 286 DG specimens. The data are expected to have many utilities, ranging from depicting the molecular characteristics of subtypes, exploring novel biomarkers, identifying prognostic signatures, and analyzing treatment-related variations. Furthermore, the data represent the largest number of diffuse glioma samples so far under the 2021 classification. These data are therefore a significant addition to global DG genome sequence databases and can be used with the new classification.

## Methods

### Specimen collection

A total of 286 glioma tissues and paired peripheral blood specimens were collected from Beijing Tiantan Hospital, Beijing Puren Hospital and Sanbo Hospital in Beijing. All patients were diagnosed with diffuse glioma by consensus, according to multiple pathological reviews by independent board-certified neuropathologists and further graded under the 2021 WHO classification.

All research was approved by the Tiantan Hospital Institutional Review Board (IRB) and performed under IRB KY2013-017-01. Written informed consent was obtained from all patients in accordance with the requirements of Beijing Tiantan Hospital Ethics Committee, and the principles of the Helsinki Declaration were carefully followed.

The specimens were frozen in liquid nitrogen within 5 min of resection. Follow-up information for each patient was also collected, including general information, survival status, clinical therapy, neuropathological classification and the requisite molecular information (Supplementary Table 1).

### Whole-exome sequencing

Genomic DNA from tumor tissue and matched blood specimens was extracted and confirmed to have high integrity by 1% agarose gel electrophoresis. The DNA concentration was measured by a Qubit® DNA Assay Kit in Qubit® 2.0 Fluorometer (Invitrogen, USA). A total amount of 0.6 μg genomic DNA per sample was used as input material for the DNA sample preparation. Sequencing libraries were generated using an Agilent SureSelect Human All Exon V6 kit (Agilent Technologies, CA, USA) following the manufacturer’s recommendations and index codes were added to each sample. The clustering of the index-coded samples was performed on a cBot Cluster Generation System using a HiSeq PE Cluster Kit (Illumina, USA) according to the manufacturer’s instructions. After cluster generation, the DNA libraries were sequenced on an Illumina HiSeq platform and 150 bp paired-end reads were generated.

### Mapping and mutation calling

**Fig. 1:**
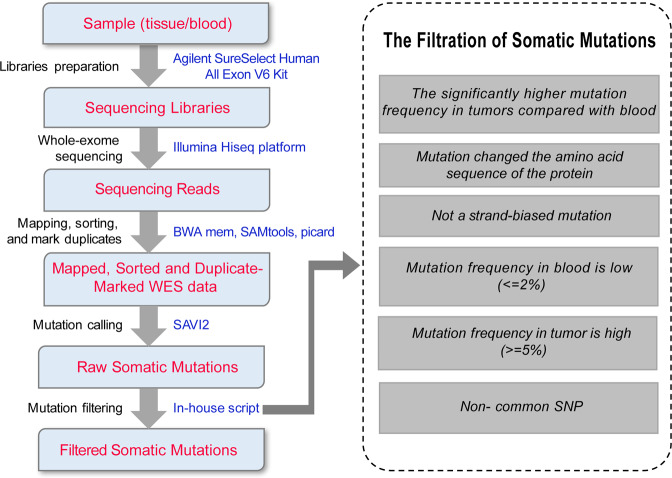
Data processing scheme. Data processing scheme from specimen preparation to mutation calling.

Whole-exome sequencing data were mapped to the hg19 genome by applying BWA software (version 0.7.12-r1039, bwa mem)^12^ with default parameters. We used SAMtools (version 1.2)^13^ (http://broadinstitute.github.io/picard/) to sort the reads by coordinates and applied Picard (version 2.0.1, Broad Institute) to mark duplicates for further analysis. An Empirical Bayesian-based tool - SAVI2 was applied to somatic mutations calling (including SNVs and short insertion/deletions) as previously described^14,15^. In this pipeline, SAMtools mpileup and bcftools were used to find variants, then the preliminary variants was further filtered out if the following criteria were met: (1) insufficient sequencing depth; (2) positions with only low-quality reads; (3) positions biased towards either strand. In particular, mutations were selected if the mutation allele frequency in the tumors was significantly higher than that in normal controls. Additionally, we used the CNVkit^16^ software to detect copy number changes. The entire flow of the processing data is illustrated in Fig. 1.

### Molecular pathological character supplementation

The *IDH1* and *IDH2* (*IDH)* mutation status was collected from pathological records and examined by pyrosequencing or immunohistochemistry with anti-IDH1 R132H antibody. *TERT* promoter (TERTp) mutation information was collected from pathological records examined by pyrosequencing. Due to the early collection of patients, some patients lacked TERTp mutation information.

*EGFR* amplification, *CDKN2A/B* homozygous deletion, chromosol 1p/19q codeletion, chromosol 7 amplification and chromosol 10 deletion were calculated by CNVkit^16^ and manually checked in Integrative Genome Viewer by two independent molecular neuropathologist.

### Relevance to TCGA and Rembrandt cohort

The Cancer Genome Atlas (TCGA) includes 916 cases of whole exome sequencing data from 401 GBMs and 515 lower-grade gliomas (LGGs), and the Rembrandt cohort includes 263 cases of SNP array data from gliomas. However, Asian patients accounted for less than 5 percent. Moreover, due to the lack of subsequent updates, the patients included could not be classified by the new classification and could not be used in the novel research system.

For our dataset, 286 Chinese glioma patients with whole exome sequencing data were included, filling the gap between these two cohorts. Our dataset could be used as an independent validation dataset for comparative analysis of TCGA, calling more focal copy number changes and covering more genetic mutations than the Rembrandt cohort.

More importantly, relying on the CGGA project, our dataset has included many newly reported molecular pathological biomarkers, and the patients can be classified by the 2021 WHO classification, providing crucial materials for global DG researchers.

## Data Records

For these 286 cases, the whole-exome sequencing (WES) data of 572 tissue and blood specimens in paired FASTQ files produced by the Illumina HiSeq platform have been deposited in the National Genomics Data Center (NGDC) under accession number HRA000071^17^. The clinical characteristics are summarized in Supplementary Table 1, including histology, WHO grade, critical molecular genetic information, sex, age at diagnosis, overall survival and current status, chemoradiotherapy, etc. Detailed clinical and molecular pathologic information is also deposited at figshare^18^. All the called SNVs per sample made by SAVI2 were deposited at figshare^19^. To classify the diffuse gliomas, IDH-wildtype NEC, IlluminaInfinium Methylation EPICBeadChip (Illumina, USA) was used to test DNA methylation information. The raw methylation data of the five samples was deposited in the GEO Expression Omnibus under the accession number of GSE216383^20^.

## Technical Validation

### Quality validation – sequencing data

**Fig. 2:**
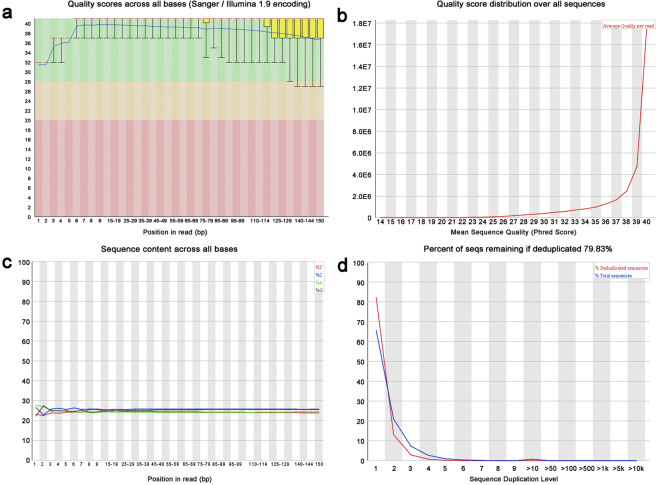
Quality control of the sequencing data (CGGA_653, forward reads). (**a**) Sequence quality scores across all bases, (**b**) quality score distribution over all sequences, (**c**) sequence content across all bases, and (**d**) sequence duplication levels.

We used FASTQC (version 0.10.1) to analyze the data quality via several measures in the FASTQ files, including a) sequence quality scores across all bases, b) quality score distribution over all sequences, c) sequence content across all bases, and d) sequence duplication levels. We selected sample CGGA_653 as a representative sample. A representative summary plot is provided in Fig. 2. Quality scores per base above 25 are considered base calls indicating high sequencing quality (Fig. 2a). The quality score distribution over all sequences was analyzed to check if a subset of sequences had universally poor quality. As a result, the average quality for most sequences was high, with scores over 30, which indicated that a significant proportion of the sequences in each run had overall high quality (Fig. 2b). Per Base Sequence Content plots out the proportion of each base position for which each of the four normal DNA bases was called. As expected, there was little difference between the different bases of each sequence run, suggesting that the library did not produce a biased sequence composition (Fig. 2c). By examining the sequence bias during polymerase chain reaction (PCR) amplification, we found that less than 5% of sequences were shown over 5 times, demonstrating low sequence duplication in the WES data (Fig. 2d).

### Fingerprint analysis of the WES samples

**Fig. 3:**
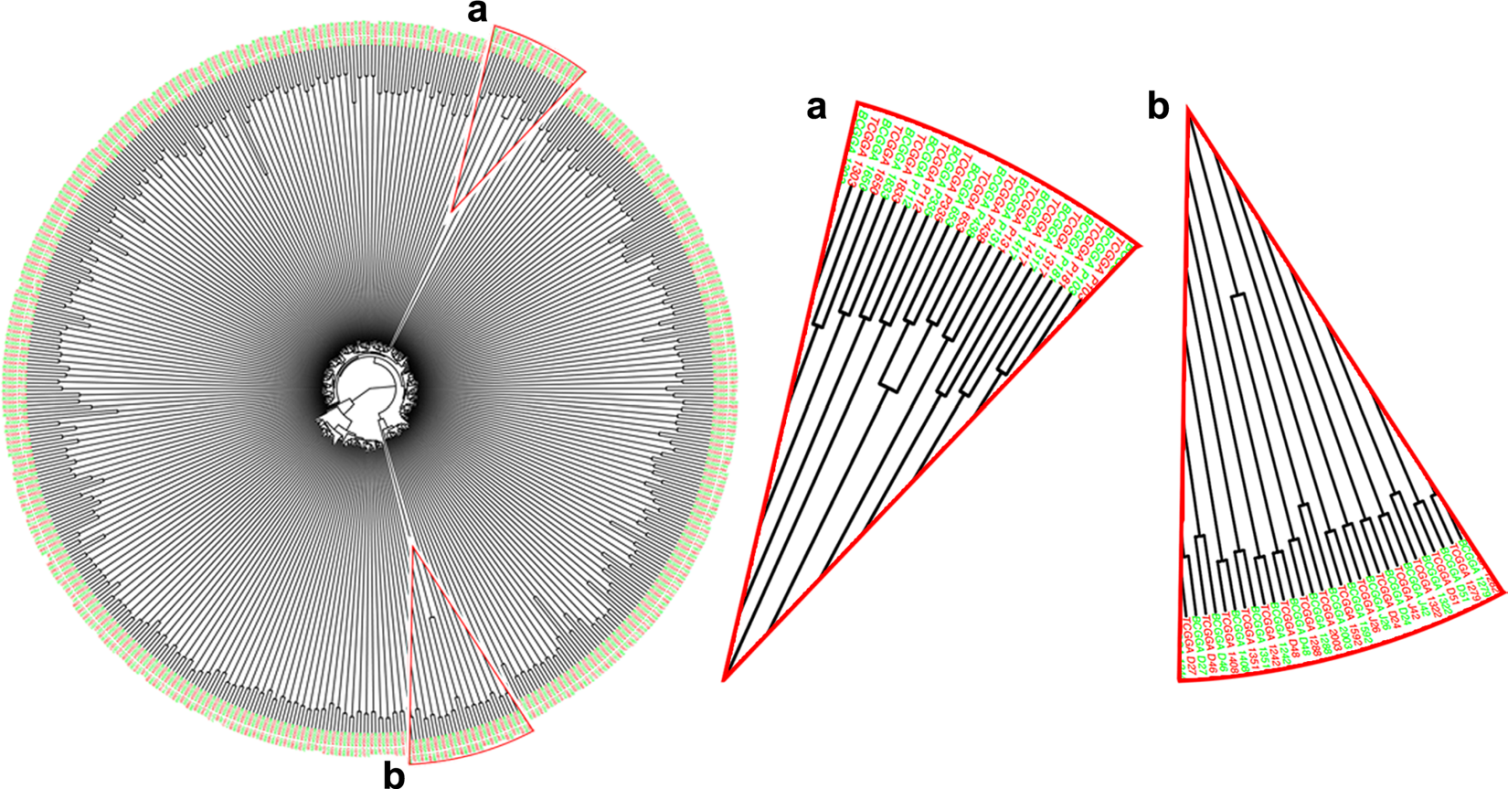
Fingerprint analysis of WES samples. Dendrogram of hierarchical clustering of 286 tumors and 286 normal samples based on Pearson correlation coefficients of SNP allele fractions. Case_ID and the tissue specimen are indicated (blood: green; tumor: red). The enlarged local views show the primary and recurrent samples from the same patient, a) CGGA_653 and CGGA_P438, b) CGGA_1288 and CGGA_2003, respectively. The correct matching of each of the 286 tumor-blood DNA pairs was determined.

Whole exome sequencing (WES) was performed on 286 glioma samples and matched blood DNA. SAVI2 was used to call germline and somatic single nucleotide variants (SNVs) and small insertions and deletions (indels) as previously described^14,15^. All the called SNVs per sample were deposited at Figshare^19^. To confirm the tumor and blood specimens from the same individual, we established a dendrogram of hierarchical clustering of tumor and blood based on known common SNP sites. Here, a common SNP is one that has at least one 1000 Genomes population with a minor allele of frequency ≥1% and for which 2 or more founders contribute to that minor allele frequency. As a result, the clustering subtree of tissue and blood samples for each individual confirmed the matching of normal blood and glioma tissue for each paired specimen (Fig. 3). In particular, since CGGA_653 (primary oligodendroglioma, CNS WHO grade 2) and CGGA_P438 (recurrent oligodendroglioma, CNS WHO grade 3) are primary and recurrent specimens from the same patient, their tumor specimens and corresponding blood specimens are clustered under a subtree. A similar phenomenon was observed between CGGA_1288 (primary astrocytoma, IDH-mutant, CNS WHO grade 4) and CGGA_2003 (recurrent astrocytoma, IDH-mutant, CNS WHO grade 4). As expected, the tumor and blood specimens from each patient were clustered together and could be used for downstream analysis.

## Usage Notes

WES has been widely used in studying genetic variations of cancers for decades. It is a powerful method to systematically depict the characteristics of tumors and discover novel diagnostic, prognostic and therapeutic biomarkers.

**Fig. 4:**
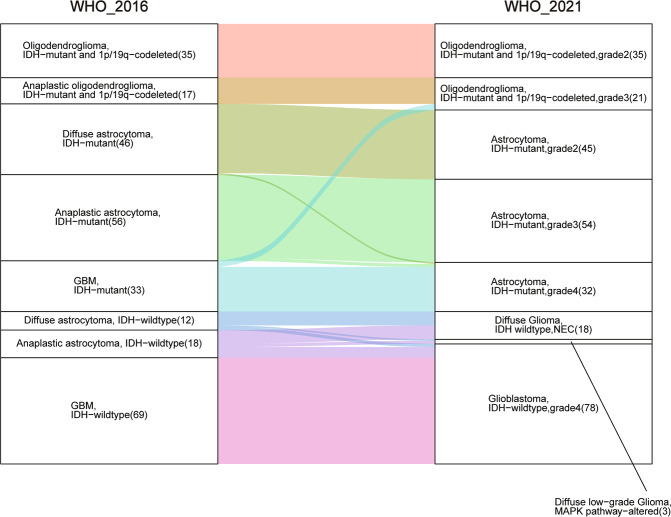
Sankey diagram of patients classified under the WHO 2021 classification. The Sankey diagram showed the changes in the WHO classification of the included patients.

**Fig. 5:**
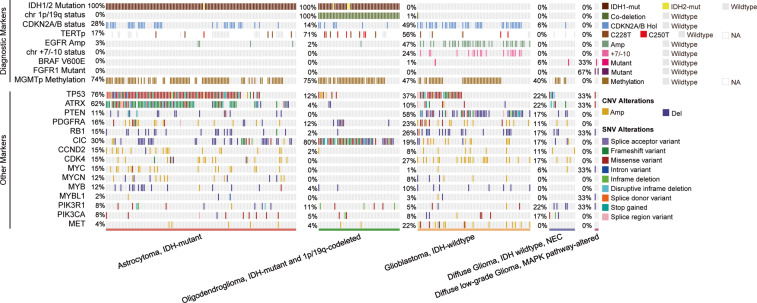
Oncoprint of collected diffuse gliomas. Characteristic mutations and copy number variations commonly occurred in specific classes.

**Fig. 6:**
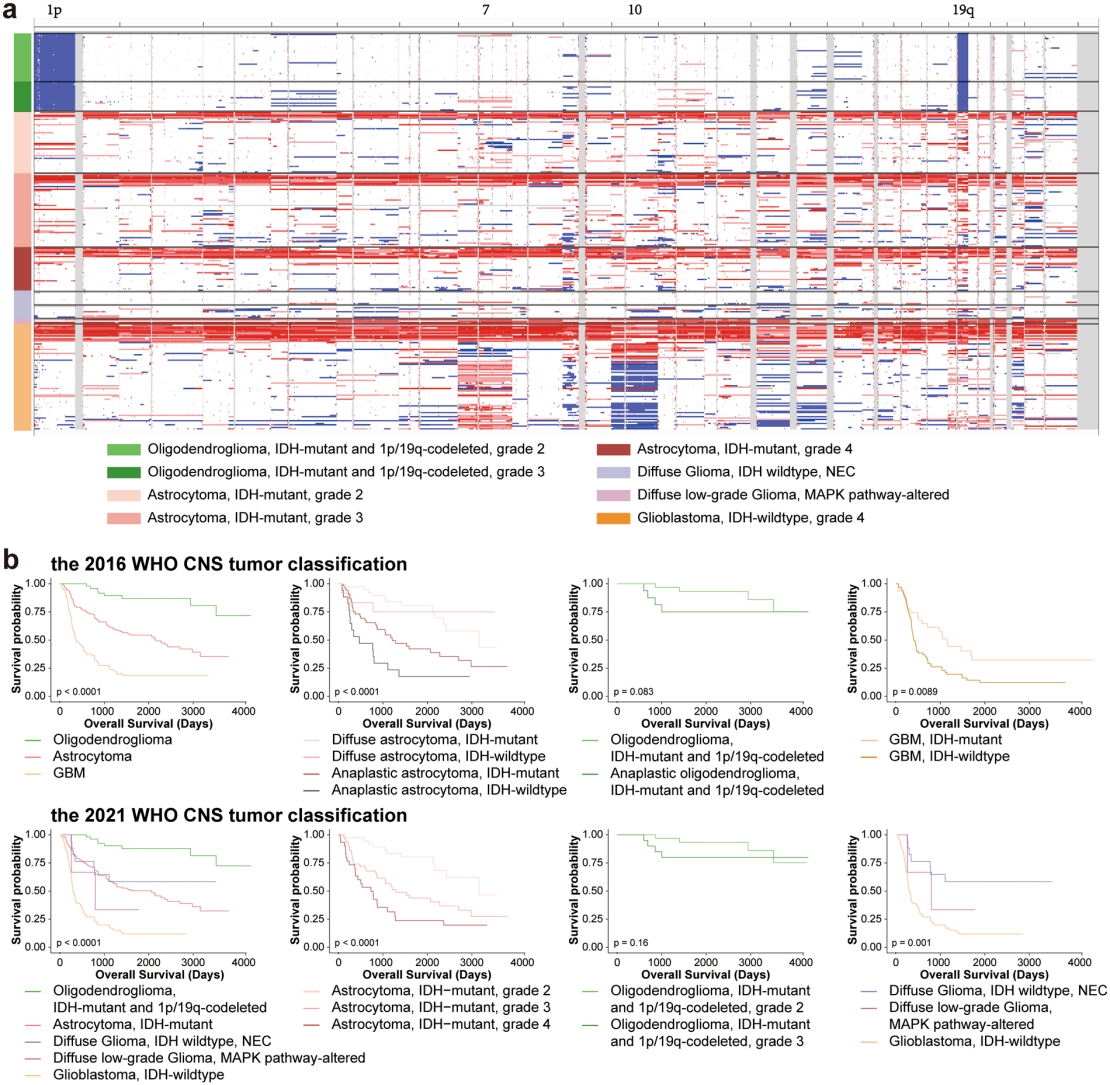
CNV plot and survival curve of different tumor types. (**a**) CNV plot of patients in different molecular subtypes. (**b**) Survival curve of patients in different molecular subtypes.

One major advantage of the data is that detailed molecular pathological characteristics have been provided and patients can be classified under the 2021 WHO Classification of Tumors of the Central Nervous System. We summarize the changes of subtype classification of patients in Fig. 4. For instance, the landscape of genetic variations of the newly classified diffuse gliomas is shown in Fig. 5. None of the “Diffuse glioma, IDH-wildtype (NEC)” cases had a TERT promoter mutation (coverage at C228T and C250T), EGFR amplification, or chromosome 7/10 alteration. Consistent with previous studies, astrocytic drivers showed frequent mutations in astrocytoma, e.g., *TP53* and *ATRX*. Correspondingly, loss of function of *PTEN*, *CDKN2A/B* deletion and *EGFR* amplification occurred commonly in glioblastomas. Additionally, the CNV plot and survival curve of different tumor types was shown in Fig. 6.

We have to acknowledge the limitation of incomplete TERTp mutation information of all patients. And relying on the CGGA project, we will continue to collect diffuse glioma samples, extend the sequencing data and supplement the molecular pathological characteristics, keeping them up to date. Additionally, we will continue updating the clinical information regularly. This clinical and molecular pathological information will help promote the further development of classification standards.

## Supplementary information

Supplementary Table 1

## Data Availability

All data analyses were completed on the Linux system using standard bioinformatic tools. The codes of the main procedures are described below. (1) **Sequencing data mapping** bwa mem -t 16 <ref_dir>/hg19.fa <sample_blood>_1.fq.gz <sample_blood>_2.fq.gz | samtools view -@ 16 -Shb -o<sample_blood>.bam samtools sort -@ 16 <sample_blood>.bam <sample_blood>.sorted picard MarkDuplicates INPUT = <sample_blood>.sorted.bam OUTPUT = <sample_blood>.sorted.dedup.bam METRICS_FILE = <sample_blood>.metrics.txt picard BuildBamIndex I = <sample_blood>.sorted.dedup.bam O = <sample_blood>.sorted.dedup.bam.bai (2) **Coordinate sorting and duplicate marking** bwa mem -t 16 <ref_dir>/hg19.fa<sample_tissue>_1.fq.gz<sample_tissue>_2.fq.gz | samtools view -@ 16 -Shb -o<sample_tissue>.bam samtools sort -@ 16<sample_tissue>.bam<sample_tissue>.sorted picard MarkDuplicates INPUT = <sample_tissue>.sorted.bam OUTPUT = <sample_tissue>.sorted.dedup.bam METRICS_FILE = <sample_tissue>.metrics.txt picard BuildBamIndex I = <sample_tissue>.sorted.dedup.bam O = <sample_tissue>.sorted.dedup.bam.bai (3) **Identification of somatic mutations** <savi_dir>/savi.py --memory 16 --superverbose --bams <sample_blood>.sorted.dedup.bam, <sample_tissue>.sorted.dedup.bam --names NORMAL,TUMOR --ref <ref_dir>/hg19.fa --outputdir <sample_dir>
